# Structural comparison of *Acinetobacter baumannii* β-ketoacyl-acyl carrier protein reductases in fatty acid and aryl polyene biosynthesis

**DOI:** 10.1038/s41598-021-86997-3

**Published:** 2021-04-12

**Authors:** Woo Cheol Lee, Sungjae Choi, Ahjin Jang, Kkabi Son, Yangmee Kim

**Affiliations:** Department of Bioscience and Biotechnology, Konkuk University, Seoul, 05029 Republic of Korea

**Keywords:** X-ray crystallography, X-ray crystallography

## Abstract

Some Gram-negative bacteria harbor lipids with aryl polyene (APE) moieties. Biosynthesis gene clusters (BGCs) for APE biosynthesis exhibit striking similarities with fatty acid synthase (FAS) genes. Despite their broad distribution among pathogenic and symbiotic bacteria, the detailed roles of the metabolic products of APE gene clusters are unclear. Here, we determined the crystal structures of the β-ketoacyl-acyl carrier protein (ACP) reductase ApeQ produced by an APE gene cluster from clinically isolated virulent *Acinetobacter baumannii* in two states (bound and unbound to NADPH). An in vitro visible absorption spectrum assay of the APE polyene moiety revealed that the β-ketoacyl-ACP reductase FabG from the *A. baumannii* FAS gene cluster cannot be substituted for ApeQ in APE biosynthesis. Comparison with the FabG structure exhibited distinct surface electrostatic potential profiles for ApeQ, suggesting a positively charged arginine patch as the cognate ACP-binding site. Binding modeling for the aryl group predicted that Leu185 (Phe183 in FabG) in ApeQ is responsible for 4-benzoyl moiety recognition. Isothermal titration and arginine patch mutagenesis experiments corroborated these results. These structure–function insights of a unique reductase in the APE BGC in comparison with FAS provide new directions for elucidating host–pathogen interaction mechanisms and novel antibiotics discovery.

## Introduction

Fatty acid synthesis is a ubiquitous mechanism, involving the provision of alkyl chains required for numerous cellular processes. In bacteria, elongation of alkyl chains by fatty acid synthase (FAS) is achieved via a series of reactions involving β-ketoacyl-acyl carrier protein (ACP) synthases (KAS), β-ketoacyl-ACP reductase (FabG), β-hydroxy-ACP dehydratases (FabA/FabZ), and β-enoyl-ACP reductase (FabI)^1^. Gram-negative bacteria rely on FAS for essential cellular processes, including phospholipid and lipopolysaccharide biosynthesis, and homoserine lactone autoinducer-based quorum-sensing^2^. As many of these processes are closely related to bacterial virulence, FAS is a potential target for the development of new antibacterial agents^3,4^.

Polyketides are secondary metabolites with diverse functions, and polyketide synthase (PKS) genes are believed to have evolved from fatty acid genes^5^. Aromatic polyketides play particularly important roles, and their biosynthesis in bacteria is mediated by the type II PKS (PKS II)^6^. This PKS requires the condensation of malonyl-ACP in an iterative manner without reduction steps to promote cyclization and functionalization^7,8^. PKS II is widely found in actinomycetes, and actinorhodin is the best studied polyketide to date. In this scheme, iterative Claisen condensation by minimal PKS involving only two keto-synthases, chain length factor-β-ketosynthase (CLF-KS) and an ACP, results in the formation of octaketides, which cyclize to yield aromatic actinorhodin via aromatase/cyclases.

Aryl polyene (APE) is a membrane lipid mainly isolated from Gram-negative bacteria. APE is the product of a biosynthesis gene cluster (BGC)^9^ that is presumed to be functionally analogous to staphyloxanthin, a virulence factor and the source of golden pigments in *Staphylococcus aureus*^10,11^*.* APE has also been suggested to play a protective role against ultraviolet (UV) irradiation in *Lysobacter enzymogenes*^12^. As APEs are frequently found in pathogens, it is tempting to presume that they play a protective role against the host immune defense response^9^. Recently, PKS II from *Streptomyces* sp. MSC090213JE08 was found to produce a polyene intermediate to generate ishigamide via a PKS II-like pathway^13^. Ishigamide is a polyene-containing amide and its limited role in reduction in PKS II has also been recognized.

**Figure 1:**
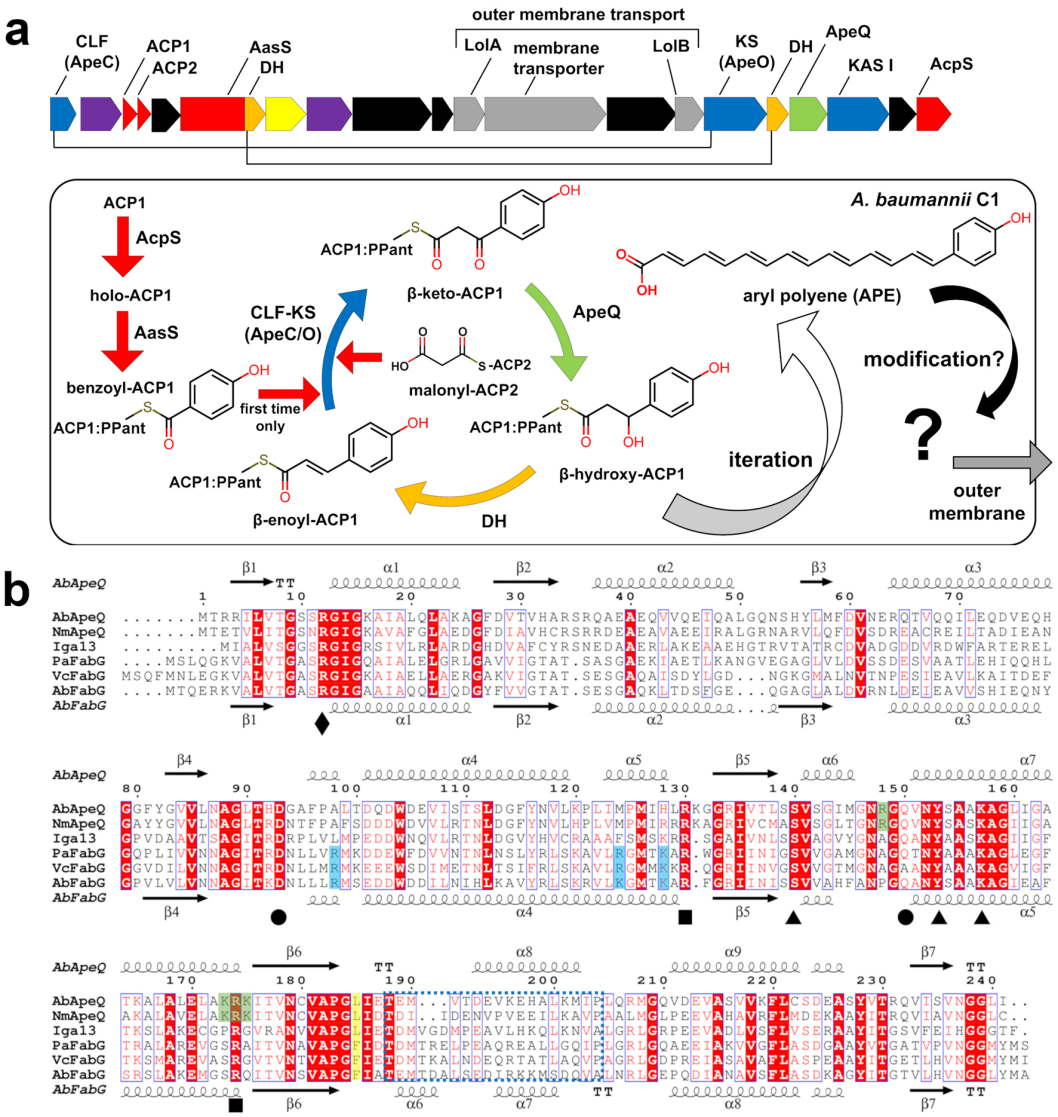
Genomic context of APE biosynthesis genes in *A. baumannii* and multiple sequence alignment of AbApeQ with its homologs. (**a**) Genomic context of *A. baumannii* APE biosynthesis genes. Proteins forming heterodimers are linked by lines. Proteins participating in APE elongation are schematically illustrated below with matching colors. *DH* dehydratase, *KS* ketosynthase, *CLF* chain length factor. (**b**) Multiple sequence alignment of AbApeQ and homologs or FabG protein: AbApeQ, *A. baumannii* ApeQ (NCBI accession number: ACC55845.1); NmApeQ, *Neisseria meningitidis* ApeQ (NP_274706.1); Iga13, *Streptomyces* sp. MSC090213JE08 ishigamide biosynthesis ketoreductase (BAX64254.1); PaFabG, *P. aeruginosa* PAO1 FabG (NP_251657.1); VcFabG, *Vibrio cholerae* O1 biovar El Tor str. N16961 FabG (AAF95169.1); AbFabG, *A. baumannii* FabG (ACC56088.1). Catalytic residues are marked by triangles. Two arginine residues that are presumed to interact with ACP and are conserved among ApeQ and FabG homologs are marked by black squares. The Leu185 residue of AbApeQ, which interacts with the 4-hydroxybenzoyl moiety, is highlighted in yellow. The Arg12 residue interacting with NADPH is marked using a diamond. The lid helices and ACP1-binding residues consisting of residues lining the ketoacyl group-binding site are indicated by a dashed blue square. Residues that appear to be important for ACP binding are highlighted in green (AbApeQ) or blue shading (AbFabG). Secondary structures of AbApeQ and AbFabG are depicted at the top and bottom of the alignment, respectively.

APE biosynthesis genes show strong resemblance to FAS, with exception of the involvement of β-enoyl-ACP reductase that produces a polyene structure. The cluster contains the CLF-KS heterodimer, which is a hallmark elongation enzyme in PKS II (Fig. 1a). The cluster also contains two distinctive ACPs in a series of open reading frames. Although the precise roles of these tandem ACPs remain unclear, ACP1 is tentatively suggested to function as a starter unit in the formation of a benzoyl-ACP conjugate, whereas ACP2 provides two carbon units in the form of malonyl-ACP^14^. This usage of discrete ACP domains is also reminiscent of PKS II (Fig. 1a). The benzoyl-ACP conjugate is generated by the acyl-ACP synthetase (AasS) protein ApeH^14^ in the presence of benzoic acid, ATP, and Mg^2+^, thereby playing a role similar to that of the benzoate:ACP ligase EncN in enterocin biosynthesis^15^. Malonyl-CoA:ACP transacylase (MCAT) is absent in the APE BGC, and the malonylation of ACP2 is presumed to be mediated by FabD, a FAS MCAT, rather than via self-malonylation^14^. Notably, the APE BGC contains a membrane transporter, periplasmic LolA-like protein, and LolB-like lipoprotein (Fig. 1a). The components of this transport system strongly suggest that the unknown product generated by the APE BGC may translocate to the outer membrane.

*Acinetobacter baumannii* is a well-known nosocomial pathogen, and the global emergence of multidrug-resistant (MDR) strains is a major concern of disease-controlling units. The FAS of *A. baumannii* has been relatively less explored compared to that of other notable pathogens such as *Pseudomonas aeruginosa* or *Mycobacterium tuberculosis*. Our previous studies focusing on the FAS proteins of *A. baumannii* led to the development of antibiotics against MDR strains^16–18^. Moreover, amino acid sequence homology analyses revealed that the majority of MDR *A. baumannii* strains harbor APE biosynthesis genes in contrast to the non-virulent standard strain.

There are two major clinical isolate groups of *A. baumannii*: global clone (GC) 1 and 2. The APE BGC is absent in *A. baumannii* strains AYE and ATCC 17978, but has been identified in all members of GC2 evaluated thus far, including *A. baumannii* strains ACICU, MDR-TJ, and BJAB07104, which are prevalent in Asia^19^. Among these, *A. baumannii* MDR-TJ, which is resistant to carbapenem, aminoglycoside, and cephalosporin antibiotics, also harbors the APE cluster (Supplementary Fig. S1)^20^; therefore, the APE cluster is presumed to be a general genotype characteristic in GC2 strains.

Studying APE biosynthesis proteins in conjunction with FAS and PKS II can have practical relevance for the discovery of drugs targeting APE biosynthesis. As the established drug target of bacterial β-enoyl ACP reductase is absent in the APE BGC, we have been searching for a FabG analog in the *A. baumannii* APE BGC (designated as ApeQ in *Xenorhabdus doucetiae*)^14^. Toward this end, in the present study, we focused on the carbapenem-resistant *A. baumannii* (CRAB) strain C1, which was clinically isolated from a patient at Korea University Anam Hospital (Seoul, South Korea). Among oxacillinase carbapenemase genes, CRAB C1 harbors the *OXA-23* gene, which is a frequently observed CRAB marker in strains circulating in the Republic of Korea^21^. Indeed, this strain is resistant to imipenem, a major carbapenem antibiotic. As a candidate FabG analog, we identified a gene that is flanked by two genes with the highest amino acid sequence homology to FabZ and a KAS protein. This provisional ApeQ protein was named AbApeQ, and its crystal structure was elucidated alone or in complex with NADPH. We also solved the crystal structure of the putative FabG of *A. baumannii* (AbFabG). As the detailed FAS genes have not yet been confirmed, we annotated AbFabG based on its genomic context, positioned between FabD and an ACP protein, which is the identical arrangement observed in *P. aeruginosa* (Supplementary Fig. S2)^22^.

To expand upon this characterization, we cloned and expressed all enzymes known to be involved in APE biosynthesis in CRAB C1, and achieved the in vitro enzymatic synthesis of APE bound to ACP1, which enabled further investigation of the impact of various mutations on the ability of AbApeQ to reduce growing APE chains. The generation of APE species can be readily monitored by UV/Vis spectrophotometry, because APE products with seven conjugated double bonds exhibit strong absorbance near 450 nm owing to their slight orange color. Such real-time analysis of APE generation can facilitate the characterization of type II PKS. Thus, the present structural analysis of APE biosynthesis proteins from *A. baumannii* combined with biochemical data provide the first glimpse of the structure–function relationships of β-enoyl ACP reductase in APE biosynthesis, along with their potential for clinical application. Specifically, our developed in vitro APE biosynthesis system can be applied to the high-throughput screening of APE biosynthesis inhibitors. This system may also be useful for assessing APE as a potential virulence factor associated with GC2 strains of *A. baumannii* and other pathogenic Gram-negative bacteria harboring the APE BGC.

## Results

### Overall structure of AbApeQ

We amplified the APE genes from the genomic DNA of CRAB C1, assuming their DNA sequences to be highly similar with those of *A. baumannii* ACICU with an established genome sequence. Sequence comparison of cloned genes from CRAB C1 showed that the APE BGC includes heterodimeric dehydratases (ApeI/ApeP) with a single active site instead of the typical homodimeric dehydratase FabZ. In CRAB C1, the protomer ApeI is fused to the C-terminus of ApeH, which forms a heterodimer with ApeP to function both as an AasS and a dehydratase (Fig. 1a). Protein BLAST identified that the highest sequence identity of ApeQ homologs of the major pathogen *Neisseria meningitidis* MC58 to AbApeQ (62%), followed by Iga13 (the ketoreductase from ishigamide biosynthesis; 41% identity) (Fig. 1b).

The apo AbApeQ crystal was confirmed as belonging to space group *P*3_1_2, with four molecules of AbApeQ in the asymmetric unit. In the Protein Data Bank (PDB), a structure (PDB ID: 6NRP) identical to ours has been deposited with no identification of function or record of publication to the best of our knowledge. The proteins are 100% identical in their amino acid sequences. The structure of the deposited protein and our predicted structure superposed with an r.m.s.d. of 0.115 Å. Here, we identified the function of AbApeQ as a β-keto-APE-ACP reductase and visualized the structure of its complex with NADPH to better understand its substrate specificity.

**Figure 2:**
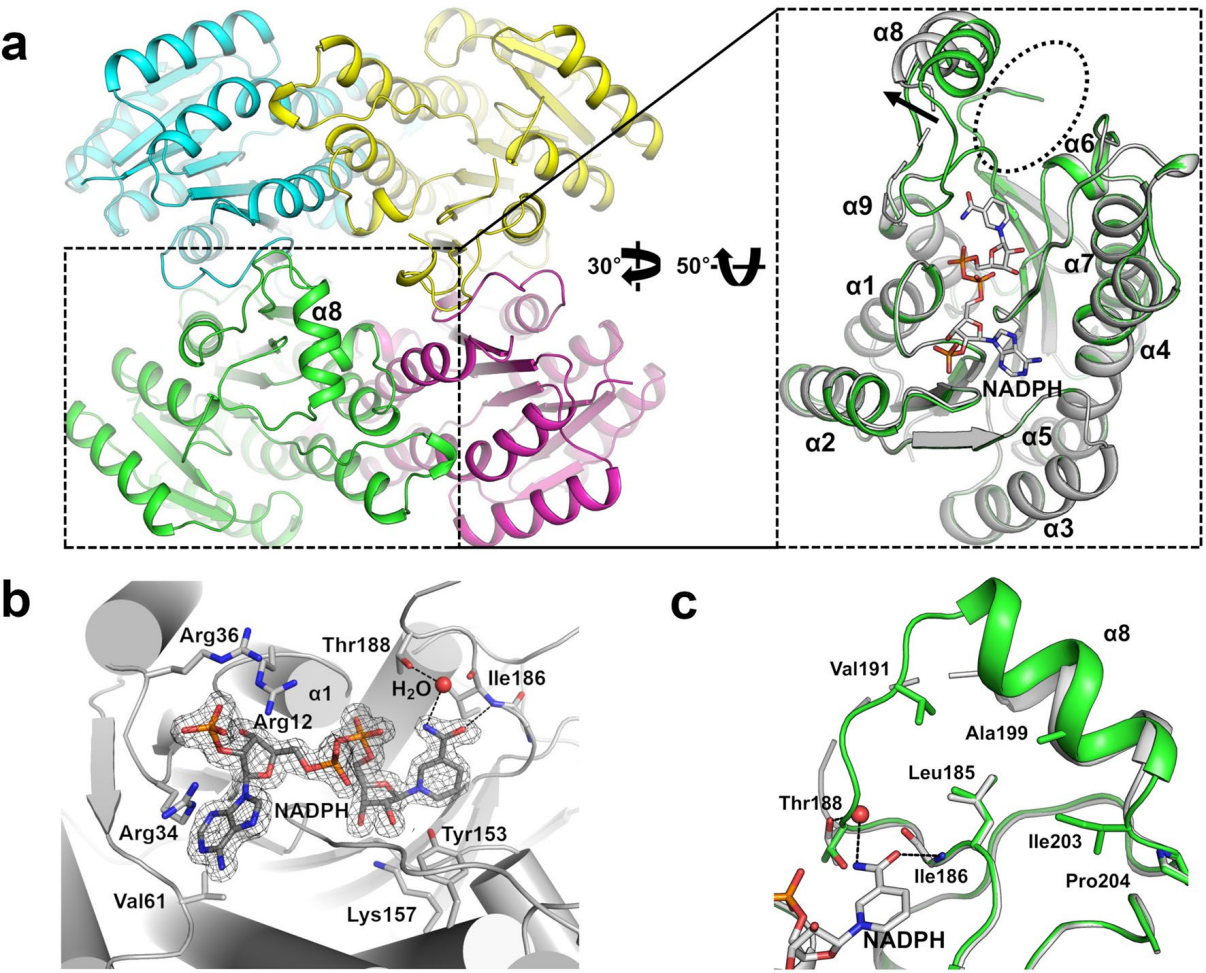
Structural changes in AbApeQ due to the binding of a substrate. (**a**) The overall structure of tetrameric apo AbApeQ. Each protomer is indicated using a distinct color. One protomer (green) is zoomed in and rotated to be superposed with AbApeQ in complex with NADPH (gray) on the right side of the panel. The movement of helix α8 in response to substrate binding is indicated by an arrow. The opening of a channel for polyene chain binding is indicated in a dashed oval circle. (**b**) Electron density for NADPH bound to the active site of AbApeQ. Residues involved in NADPH binding are shown as stick models; active-site residues (Tyr153 and Lys157) are also shown. A water molecule mediating the interaction between NADPH and Thr188 is rendered in a sphere model. (**c**) Detailed view of the flap structure consisting of helix α8 in apo ApeQ (green) or ApeQ-NADPH complex (gray) structures. Residues interacting with Leu185 are presented as stick models. A water molecule mediating the Thr188–NADPH nicotinamide interaction is rendered as a sphere.

The overall structure of AbApeQ shows the typical Rossman α/β fold protein structure. The four molecules are related by the point group of 222 to form a tetramer (Fig. 2a). Due to the high temperature factors of the substrate-binding lid (residues Thr188‒Pro204 in Fig. 1b), some residues could not be modeled. Comparison of the amino acid sequence of AbApeQ with the ApeQ homolog from *N. meningitidis* MC58 (NmApeQ) and FabG from *E. coli* (EcFabG) demonstrated homology over the latter half of the ApeQ sequences, including α7 and α9, where the tetrameric interfaces are located (Figs. 1b, 2a).

Further comparison of the linker region leading to α8, which houses the binding channel for the substrate β-keto group, showed that the loop is 2–3 residues shorter in ApeQ proteins relative to that of FabG proteins (Fig. 1b). Helices α2, α3, and α4, which are distant from the active site and exposed to the solvent, constitute the least conserved regions among ApeQ proteins (Fig. 2a). One dimeric interface forms extensive—mostly hydrophobic—interactions via α7 and β6. The other dimeric interface occurs through α5 and α7, also with hydrophobic interactions. Both dimeric interfaces are extensive, such that the quaternary structure may remain stable in the aqueous phase, which was confirmed using size-exclusion chromatography (Supplementary Fig. S4). The apparent molecular weight was 142 kDa, which is much higher than the calculated molecular weight of 105.6 kDa. We assume this discrepancy is because of the planar shape of the tetramer.

### Conformational change due to NADPH binding

We obtained the structure of the AbApeQ–NADPH complex by co-crystallization of AbApeQ and NADPH. The crystal was in the *P*2_1_ space group; it contains four AbApeQ molecules in the asymmetric unit, as found for the apo AbApeQ crystal. We observed highly flexible residues spanning positions 189–194, a loop, and the N-terminus of helix α8, some of which could not be modeled (Fig. 2c). The disordered structure of this helix region, which coincides with the lid structure for substrate binding, has been observed in other FabG structures in complex with NADPH^23,24^. The unambiguous electron density for NADPH was determined (Fig. 2b). The adenine ring is stacked between Arg34 and Val61, whereas the Arg12 residue interacts with 2′-ribose phosphate. The diphosphate moiety is stabilized by a dipole moment from helix α1. The primary amide of the nicotinamide group is within 3 Å from a hydrogen of a water molecule that is bound to Thr188 and the main chain amide of Ile186 (Fig. 2b). Two catalytic residues in FabG, Tyr153 and Lys157, are present in the vicinity of NADPH^23^. Only two molecules, a dimer through α4 and α6, showed NADPH-like electron density (Fig. 2b). The other dimer was devoid of any electron density that could be discernable as that of NADPH except for the weak density of 2′-ribose phosphate (data not shown). The phosphate moiety is stabilized by three arginine residues, Arg12, Arg34, and Arg36 (Fig. 1b), whereas only Arg12 is conserved in FabG proteins^25^.

A previous report demonstrated that binding of the substrate NADPH to *E. coli* FabG (EcFabG), particularly the specific interaction involving a nicotinamide group, causes movement of the helix corresponding to α8 in AbApeQ, which then facilitates binding of the second substrate, β-ketoacyl ACP^23^. A similar conformational change was observed for AbApeQ, in which the nicotinamide group of NADPH forms hydrogen bonds with Thr188 through a water molecule (Fig. 2b). Consequently, α8 swings by approximately 13°, with Pro204 serving as the hinge site (Fig. 2c). This shift in the orientation of α8 was observed in all chains, regardless of the presence of NADPH (Fig. 2c). However, in the absence of NADPH, the side chains of Val191, Ala199, Ile203, and Leu185 form a shallow hydrophobic core. NADPH binding then causes the Thr188 backbone atoms to flip, which disrupts the hydrophobic network to expose Leu185 to the binding site (Fig. 2c).

### Crystal structure of the β-ketoacyl ACP reductase AbFabG

As a member of Gammaproteobacteria, *A. baumannii* is closely related to *P. aeruginosa* and *E. coli*. In particular, *P. aeruginosa* and *A. baumannii* have similar FAS genes, whereby the host fatty acids can be incorporated for de novo fatty acid synthesis^17,26^. FabG of *P. aeruginosa* (PaFabG) was identified as a potential drug target with allosteric inhibition targeted to multimeric interfaces^24^. Structural and functional analyses of *Vibrio cholerae* FabG (VcFabG) were also reported^27^. The amino acid sequence of AbFabG shows 59% and 58% identity with those of VcFabG and PaFabG, respectively (Fig. 1b). We determined the crystal structure of AbFabG at a 1.79 Å resolution. We also attempted to crystallize the AbFabG–NADPH complex. However, the obtained crystals diffracted poorly and were not adequate for high-resolution structure analysis. The apo AbFabG crystal structure demonstrated the presence of four molecules of AbFabG in the asymmetric unit.

**Figure 3:**
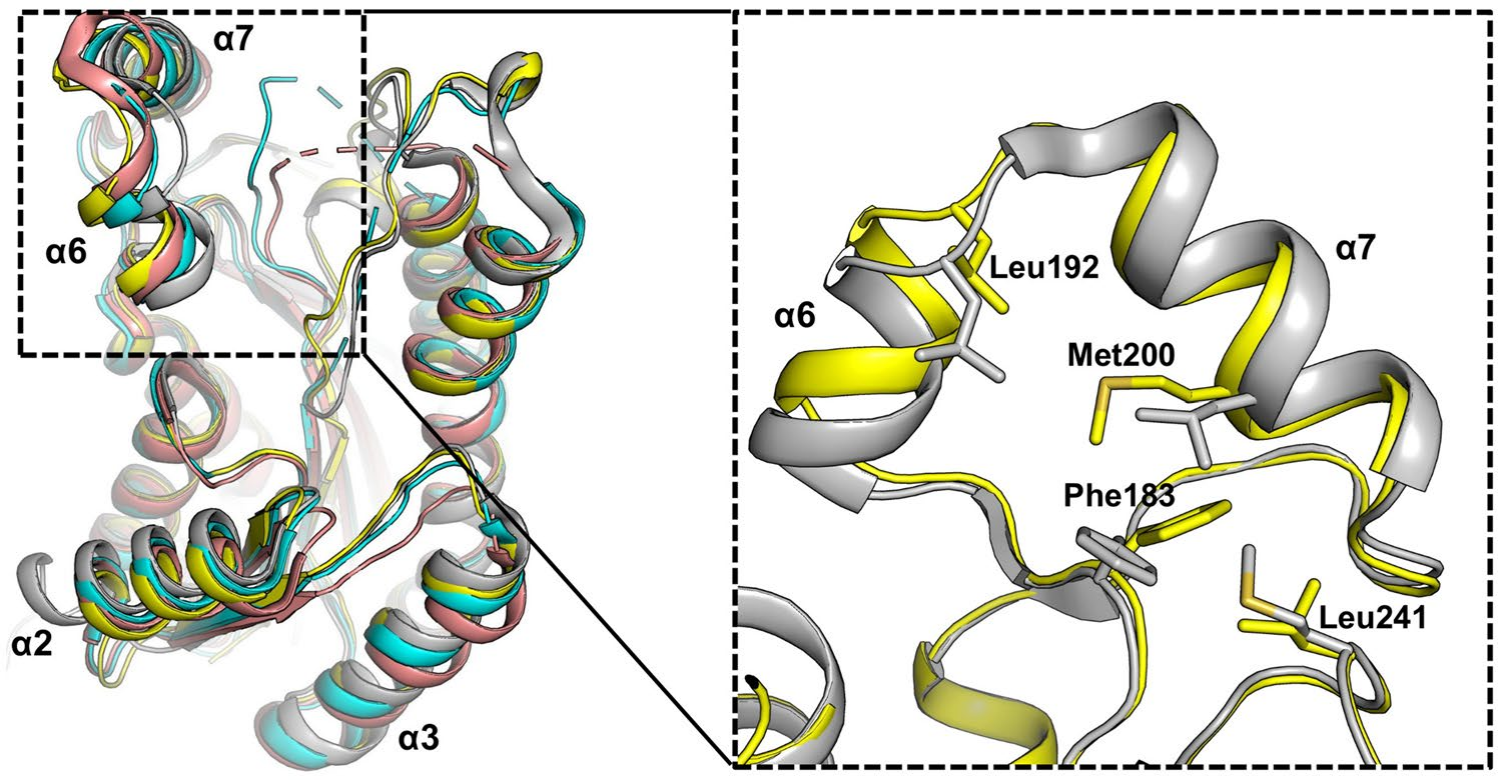
Structures of AbFabG (yellow), *E. coli* EcFabG (cyan, PDB ID: 1I01), *P. aeruginosa* PaFabG (gray, PDB ID: 4AFN), and *V. cholerae* VcFabG (salmon, PDB ID: 5END) are superposed. In the blown-up view, oriented in the same viewpoint as in Fig. 2c, AbFabG (yellow) and PaFabG (gray) are used to illustrate the residues (stick models) that are compared in the text.

To assess the structural similarity, several apo FabG crystal structures were superposed (Fig. 3). EcFabG, PaFabG, and VcFabG structures were superposed onto the AbFabG structure with a root mean square deviation of 0.74, 0.58, and 0.54 Å, respectively. Other than four helices (α2, α3, α6, and α7), the overall structures superposed well (Fig. 3). In the flexible lid, the central hydrophobic residue—Phe183 in AbFabG and Phe186 in PaFabG—undergoes a variable conformation depending on the neighboring residues (such as Leu192, Met200, and Leu241 of AbFabG), resulting in variation of the orientation in α7 or α8 (Fig. 3). Comparison of the AbApeQ and AbFabG structures (Fig. 2b vs. Fig. 3) revealed a prominent difference at the point where the lid helices cover the substrate-binding channel. In AbFabG, there is a helix in place of the loop β6-α8 in AbApeQ. This is because the loop β6-α8 of AbApeQ is shorter than that of AbFabG by 2–3 residues (Fig. 1b). The superposed ribbon models of AbApeQ and AbFabG, with these differences annotated, are presented in Figure S7.

### Surface electrostatic potential difference between AbApeQ and AbFabG

**Figure 4:**
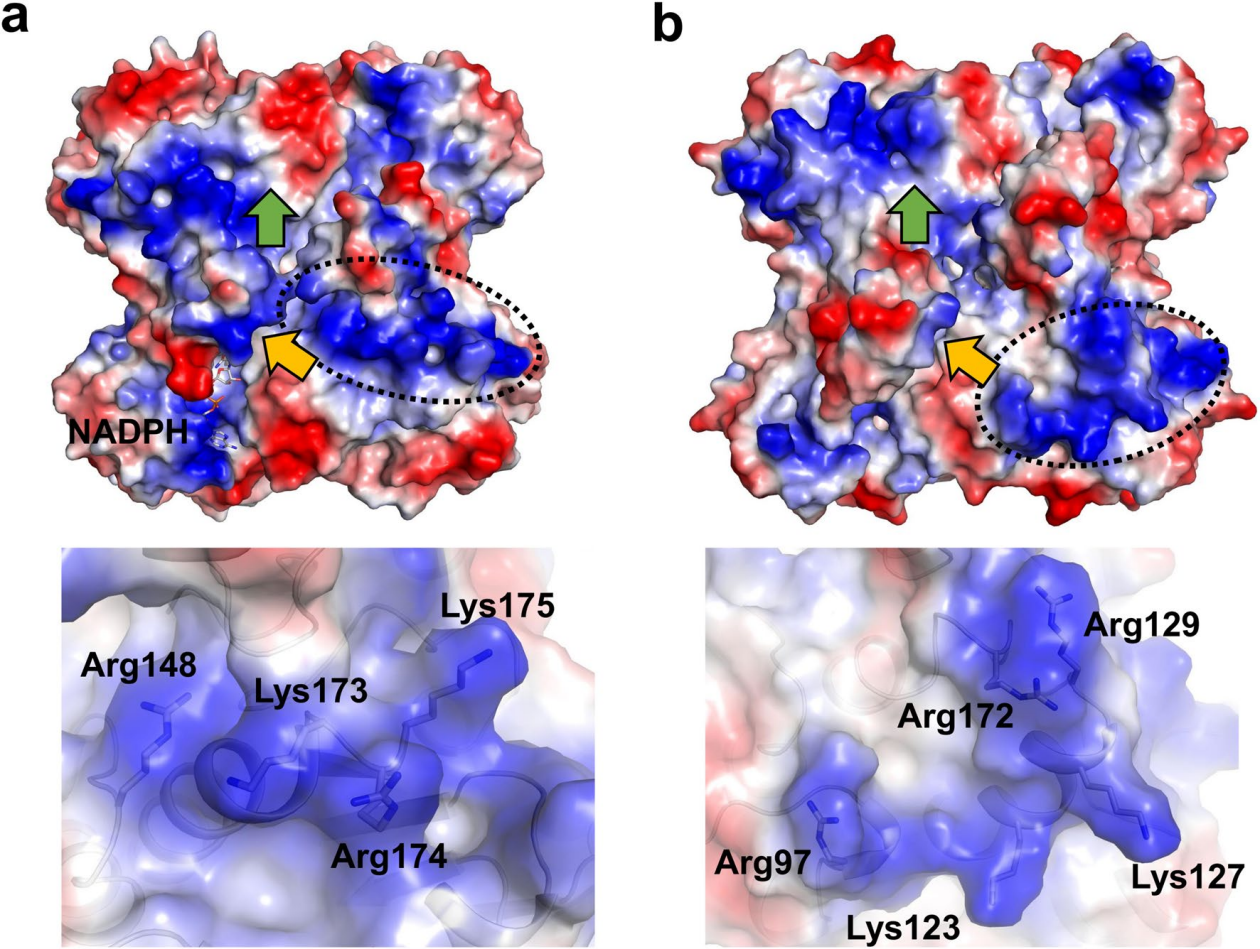
Electrostatic potential surface plot of the AbApeQ–NADPH complex (**a**) and AbFabG (**b**) tetramers. Entrance and exit of the PPant:APE substrate moiety are indicated by yellow and green arrows, respectively. Each surface with a dashed oval is blown up in each bottom panel, in which the residues presumed to interact with the respective substrate ACP are presented in stick models and labeled.

The surface electrostatic potential profiles of the ApeQ and FabG structures exhibited a clear difference at the presumed ACP1-binding site (Fig. 4). This difference was also reflected in the multiple amino acid sequence alignment (Fig. 1b). In previous studies, the interaction between ACP and FabG proteins was attributed to electrostatic interactions^28^. ACPs are acidic proteins, and residues on helix II have been found to be important for binding to counterpart biosynthesis enzymes. In ApeQ structures, three arginine residues (Arg130, Arg148, and Arg174) are closer to the substrate-binding lid (Fig. 4), whereas positively charged residues in AbFabG (Arg97, Arg123, and Arg129) showed a different electrostatic potential distribution in separate selection. Combined with surface electrostatic potential and orthogonal cognate ACP1, AbApeQ is specialized for generating only the APE product in contrast to AbFabG.

### FabG cannot be substituted for ApeQ in APE biosynthesis

**Figure 5:**
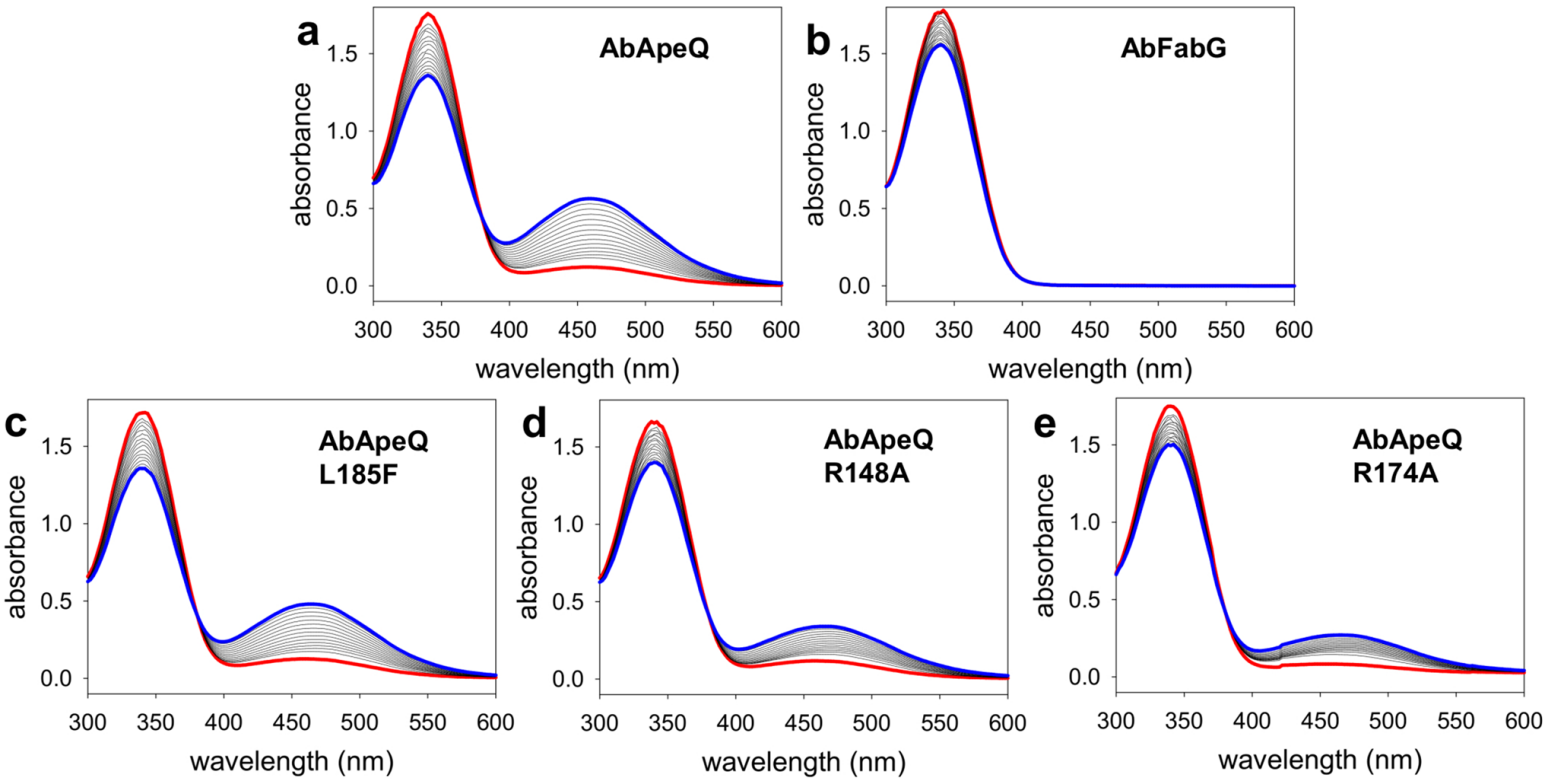
In vitro assay of APE biosynthesis. The cumulative spectrum of the in vitro APE biosynthesis product was recorded every 60 s over a period of 900 s. The initial and final spectra are rendered in thick red or blue lines, respectively, following the reaction with (**a**) AbApeQ, (**b**) AbFabG, (**c**) AbApeQ L185F, (**d**) AbApeQ R148A, and (**e**) AbApeQ R174A.

The UV/Vis spectra of APE-ACP1 were measured to compare the activity of AbApeQ or AbFabG in the context of APE biosynthesis. All enzymes required for the generation of APE-ACP1 were purified and used for the in vitro assay (Fig. S5). Early in the reaction, the products displayed absorbance at 450 nm, which increased to 460 nm toward reaction completion (Fig. 5a). This bathochromic shift is presumably due to the presence of intermediates with fewer double bonds, which stems from the iterative nature of APE biosynthesis. In contrast to AbFabG, which showed almost negligible generation of APE-ACP1 (Fig. 5b), the reaction with AbApeQ resulted in the rapid generation of APE species with a pronounced peak near 460 nm, revealing that only ApeQ can contribute to APE biosynthesis. Even without noticeable generation of APE species, the reaction by AbFabG resulted in a slow decrease in NADPH absorption, suggesting that NADPH is nonetheless consumed, although the reason is currently unclear. The assay was repeated with oxygenated buffer in the absence of enzymes. The decay of NADPH was negligible, which indicated that the oxygen in the buffer is not the reason for the background consumption.

### Leu185 and the arginine patch contribute to substrate binding

In multiple sequence alignment, the lid helices of AbApeQ were found to be three residues shorter than those of AbFabG (Fig. 1b). The same lid for KR in aromatic PKS, such as actKR, is even more rigid, with two long helices^29,30^. Thus, the first helix of the two-helix flap structure for FabG or actKR is missing in ApeQ (Fig. 1b). Leu185 resides in the center of this lid in AbApeQ. To verify the importance of this particular residue with respect to the specificity of APE biosynthesis, we prepared an L185F variant of AbApeQ to mimic Phe183 in AbFabG, which reduced the generation of APE (Fig. 5c), suggesting a contribution of Leu185 to APE biosynthesis. ACPs are negatively charged proteins, and their recognition by positively charged residues in FAS or PKS is frequently observed. For example, Arg172 is implicated in ACP binding by *E. coli* FabG^31^. In line with these previous findings, we found that the corresponding residue in AbApeQ, Arg174, is conserved, which is positioned between two basic residues: Lys173 and Lys175 (Fig. 1b). Arg148 is also located to allow for the formation of an arginine patch with Arg174. To determine the relevance of these basic residues in ACP1 binding, we prepared R148A and R174A mutants and measured their activities (Fig. 5). The R148A mutant showed reduced activity and that of R174A was even further reduced, suggesting that these residues are critical for substrate binding. From the spectral data, the time course activity at 460 nm was extracted and charted to assist with the comparison (Figure S6).

**Figure 6:**
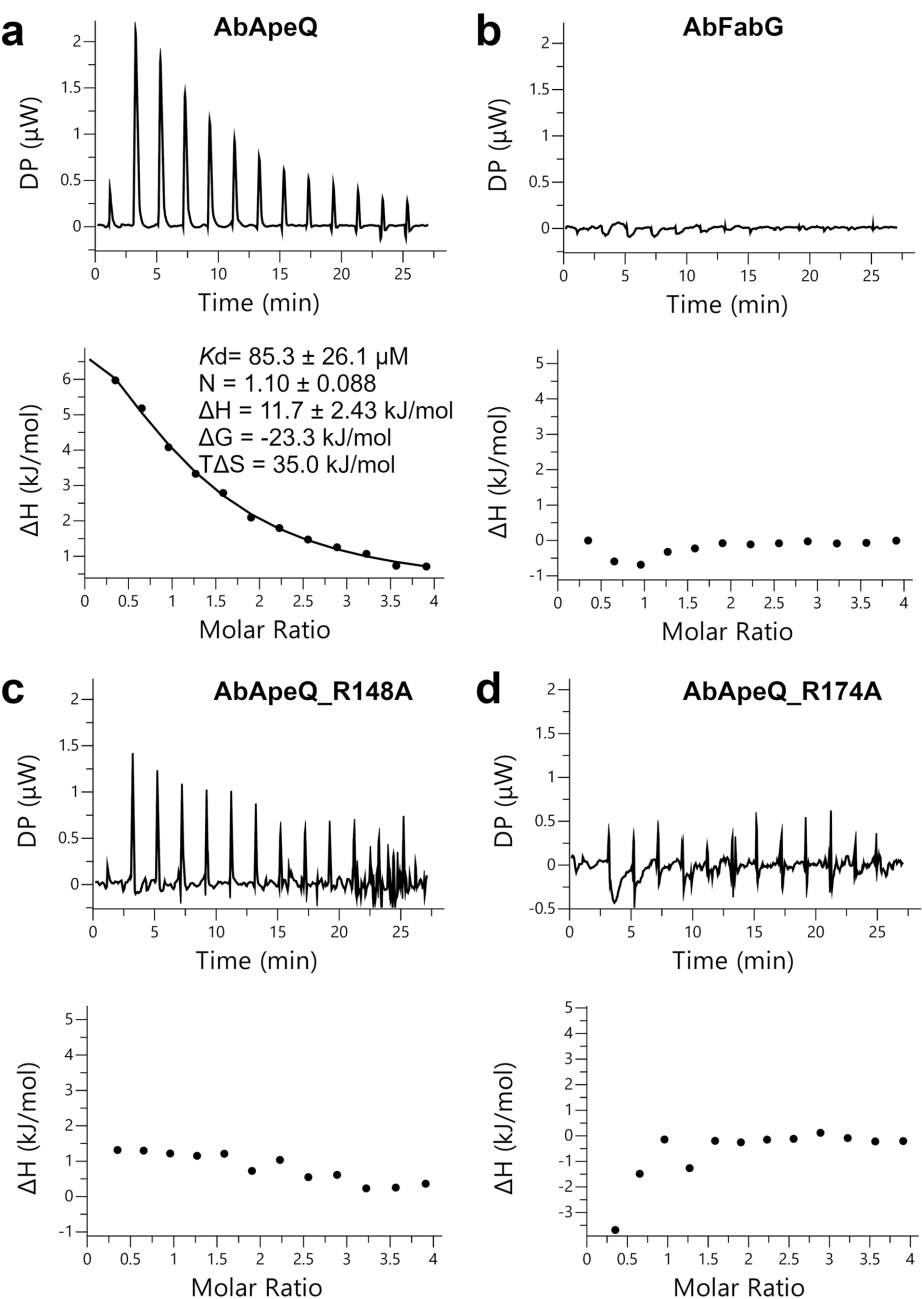
Isothermal titration of AbFabG and AbApeQ or its variants by holo ACP1. (**a**) AbApeQ, (**b**) AbFabG, (**c**) AbApeQ R148A mutant, (**d**) AbApeQ R174A mutant. Upper panels show raw data of heat changes during titration. Lower panels exhibit integrated heat changes. Only the titration of AbApeQ could be fitted with a model, for which the calculated *K*d is also shown.

Isothermal titration (ITC) of AbApeQ and cognate ACP binding showed that AbApeQ and holo ACP1 exhibited 1:1 stoichiometry (N sites = 1.11) binding with a *K*d of ~ 80 μM (ΔH = 3.05, ΔG = − 6.18, and TΔS = 9.23 kcal/mol) (Fig. 6a). By contrast, AbFabG failed to bind ACP1 (Fig. 6b). Moreover, the thermogram for the AbApeQ R148A mutant displayed weak binding (Fig. 6c); however, we could not measure the Kd for this mutant in the same binding condition as AbApeQ. The R174A mutation also resulted in a nearly negligible binding thermogram (Fig. 6d). The *K*d value for AbApeQ is higher than that reported for a ketoreductase in mycolactone biosynthesis^32^.

## Discussion

Ketoreductases from PKS are evolutionarily related to β-ketoacyl-ACP reductases^21^. Therefore, it is reasonable to hypothesize that the polyene structure of the growing polyketide chain synthesized by PKS is similar to that of the chains synthesized by FAS, suggesting that ApeQ likely evolved from FabG to accommodate the bulkier substrate of polyene. A conjugated structure of polyenes shows potent biological reactivity toward biological membranes; accordingly, numerous antifungal agents from bacteria are derived from macrolides with polyene groups. Moreover, polyenes can scavenge reactive oxygen species, thereby allowing the bacteria to evade host immune responses. However, APE biosynthesis has been relatively less explored when compared to the extensive studies on the polycyclic, aromatic compounds resulting from PKS. In the genome of the GC2 strain *A. baumannii* ACICU, the APE BGC lies in the region corresponding to the sequence between the ribosome-associated GTPase *EngA* and the *AcpS* loci of GC1 strain *A. baumannii* AYE. This suggests that the APE phenotype is relatively recently acquired via an unknown mechanism (Supplementary Fig. S3). In the context of pathogenicity, APE may be involved in host–pathogen interactions, thereby contributing to infection. Indeed, the unknown product containing APE is presumed to translocate to the outer membrane in MDR *A. baumannii*. Thus, exploration of the selective advantage of APE biosynthesis acquired by GC2 strains over other MDR *A. baumannii* strains such as GC1 strains could provide new insights for understanding the pathogenicity of MDR A*. baumannii* and developing new treatments.

In the calicheamicin synthesis pathway, a polyene of 1,3,5,7,9,11,13-pentadecaheptaene is the intermediate synthesized by the polyketide synthase CalE8, which is required for subsequent cyclization to form the enediyne moiety^33^. CalE7 is a hot dog fold-containing thioesterase that is required for the release of polyene; a crystal structure was used to build a computational binding model of a polyene substrate^33^, and a crystallographic model of substrate binding of the dynemicin biosynthesis thioesterase DynE7 was also reported^34^. Binding of the polyene moiety was accompanied by an induced fit near the entrance due to the rigid nature of the substrate. Similar adjustment of the active-site channel would therefore be necessary for ApeQ to accept APE precursors. Our structural analysis and comparison suggest that the Leu185 residue of AbApeQ is located at the site of initial benzoyl moiety binding compared with the Phe183 residue of AbFabG. Thus, this residue functions as a gateway of substrates, thereby facilitating the imminent processing of growing APE chains. The combination of aryl-binding Leu185 and a unique arginine patch seems to facilitate the recognition of cognate benzoyl-ACP1 by AbApeQ, leading to growth of the APE chain, which makes AbFabG incompetent as an APE reductase.

In contrast to the generic reaction products of FabGs, the reactions catalyzed by PKS II ketoreductases are specific to their own specific products. Furthermore, the reduction step is often coupled to the spontaneous cyclization reaction, which occurs in a cyclization chamber^7^. Given the FAS–PKS hybrid nature of the APE BGC where ApeQ resides, it would be interesting to compare the active site of ApeQ with those of other PKS II ketoreductases. Among them, the structures of actinorhodin (actKR), hedamycin (hedKR), and two angucyclinone-6 biosynthesis ketoreductases (UrdMred and LanV) have been determined to date^30,35,36^. Structural analysis of actKR revealed that ketoreductases in PKS II show a distributed binding site for acylated holo-ACPs by separately binding to ACP at the phosphopantetheine (PPant) moiety and the growing polyketide chain^35^. The α7 region of actKR, where the ketoreductases show the least homology, is important for the growing arms of the polyketide^37^. Assuming that AbApeQ follows the same scheme as actKR, two arginines (Arg130 and Arg174) at the interface of ACP1 and AbApeQ will provide the first contact between the reductase and substrate, thereby allowing for the presentation of *N*-acetylcysteamine, followed by the polyene arm.

However, the detailed binding mode of polyene in ketoreductases has not been studied until now. In this study, the processivity of the elongated APE chain was observed based on the bathochromic shift of spectrum peaks. In PKS, the chain lengths of polyketides cannot be easily tracked owing to the limited discrimination of intermediates with varied lengths. There was a prevalence of shorter APE-ACP species in APE early in the reaction, as observed by the shift of the peak in the UV/Vis spectrum, which could be measured readily, although in a qualitative manner. We acknowledge that the identification methods used may seem unsatisfactory and not very rigorous due to the lack of analytical methods such as LC–MS; however, we will pursue this further in our subsequent studies.

Moreover, our structural analysis of β-ketoacyl reductases involved in APE and fatty acid biosynthesis suggest a specific interaction between β-keto ACP reductase and ACP. Cognate ACP1 from the APE BGC appears to be required for the proper generation of APE products. This is reminiscent of the situation in RhlG, a reductase involved in rhamnolipid biosynthesis. Although the requirement of RhlG in rhamnolipid biosynthesis is still debated, its crystal structure suggests that an as-yet-identified ACP, distinct from the FAS ACP, will be needed for proper interaction based on its unique surface electrostatic potential^38^. Surprisingly, we found a difference in the surface electrostatic potential between AbApeQ and AbFabG. Similarly, our structural and biochemical analyses of AbApeQ revealed that this reductase is specifically involved in APE biosynthesis and requires a dedicated ACP for its proper functioning. Two tandemly translated ACPs are found in every APE BGC, and similar to RhlG, a unique ACP1 may be a prerequisite for ApeQ activity. One of the reasons that the APE BGC harbors a unique set of ACPs that is distinct from the FAS counterpart may be to limit the cross-activity between FAS and APE biosynthesis intermediates.

**Figure 7:**
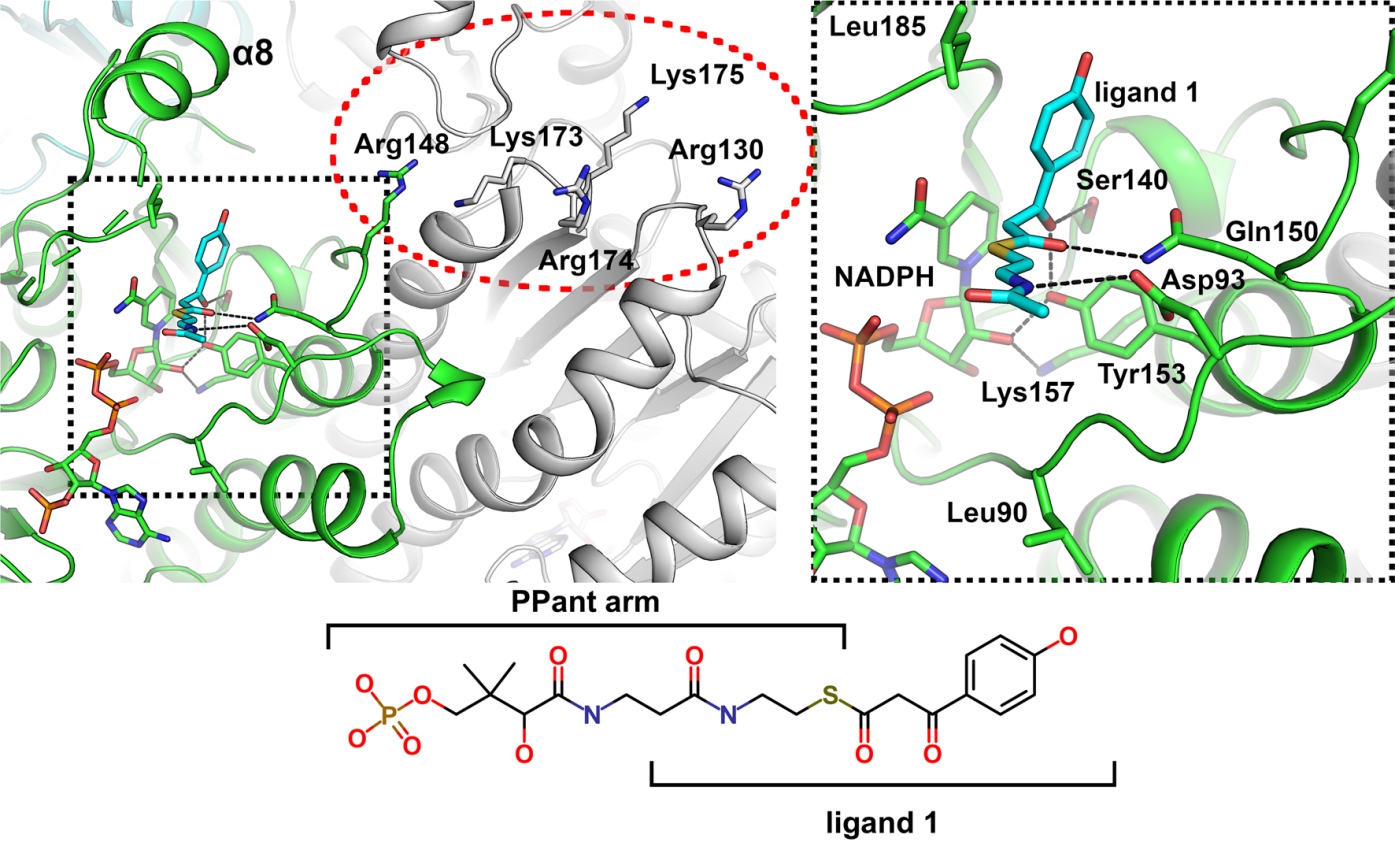
Structural model of AbApeQ–substrate interactions. Substrate-binding sites for 4-hydroxybenzoyl-holo-ACP1. Charged surface residues are marked by a red dotted oval and rendered in stick models and labeled. A tetrameric partner of AbApeQ protomer is rendered in gray. The model of the 4-hydroxybenzoyl-β-ketoacyl group (ligand 1) bound to the active site is shown as a cyan stick model. The structure of the PPant arm with the 3-(4-hydroxyphenyl)-1,3-dioxopropane moiety is depicted. The docked model dubbed ligand 1 is specified. The substrate-binding pocket is in a dashed box and the detailed interaction with the active-site residues is rendered in the right panel.

To understand the binding of the growing APE chain into the active site, we modeled the 3-(4-hydroxyphenyl)-1,3-dioxopropane moiety with the N-acetylcysteamine group from PPant arm (ligand 1) into the active site of ApeQ (Fig. 7). We modeled the substrate such that the β-keto group was hydrogen bonded to Ser140, a member of the conserved Ser-Lys-Tyr triad in the SDR family (Fig. 7). In contrast to FAS β-ketoacyl reductase, in which a fully oxidized alkyl chain is generated, stereochemistry in APE biosynthesis is important, since conjugated *trans*-double bonds should be generated for the final product (Fig. 1a). Similar to PKS, APE β-ketoacyl reductase should have a defined substrate-binding mechanism for this stereochemistry. For the *trans*-double bond conjugated system, KR should provide hydrogen from the nicotinamide of NADPH to form a *D*-hydroxy group. Based on a previously proposed model of B-type KR, MinKR6^39^, we modeled Asp93 and Gln150 to interact with the amine group of the amide bond and ketone from the thioester group of substrate, respectively (Fig. 7). The Gln150 and Tyr153 pair orients the substrate to present its proper side to the nicotinamide of NADPH. Asp93 is in the same loop as the LDD motif, albeit a feature in KR from type I PKS, from B-type ketoreductases and also appears to be important for *D*-hydroxy group generation in PKS ketoreductases^40^. Thus, Leu90-Asp93 may be a key determinant in the B-type trait in FAS-like ketoreductases. Interestingly, Leu185, the residue conserved among ApeQ homologs, is poised to form a direct interaction with the benzoyl group. The bulkier sidechain of Phe183 in AbFabG will impose greater hindrance to the growing alkyl chain. Given this steric hindrance, the presence of leucine at this position can be assumed to be less of a hindrance than phenylalanine, which is conserved in FabG proteins. Iga13 is also a KR from a polyene ishigamide biosynthesis type II PKS in *Streptomyces* sp. MSC090213JE08^41^. The ishigamide is lacking an aryl group, and therefore, the presence of Leu182 in the same position as Leu185 of AbApeQ is intriguing. However, the presence of leucine at this position could possibly be a determinant of polyene biosynthesis KRs.

**Figure 8:**
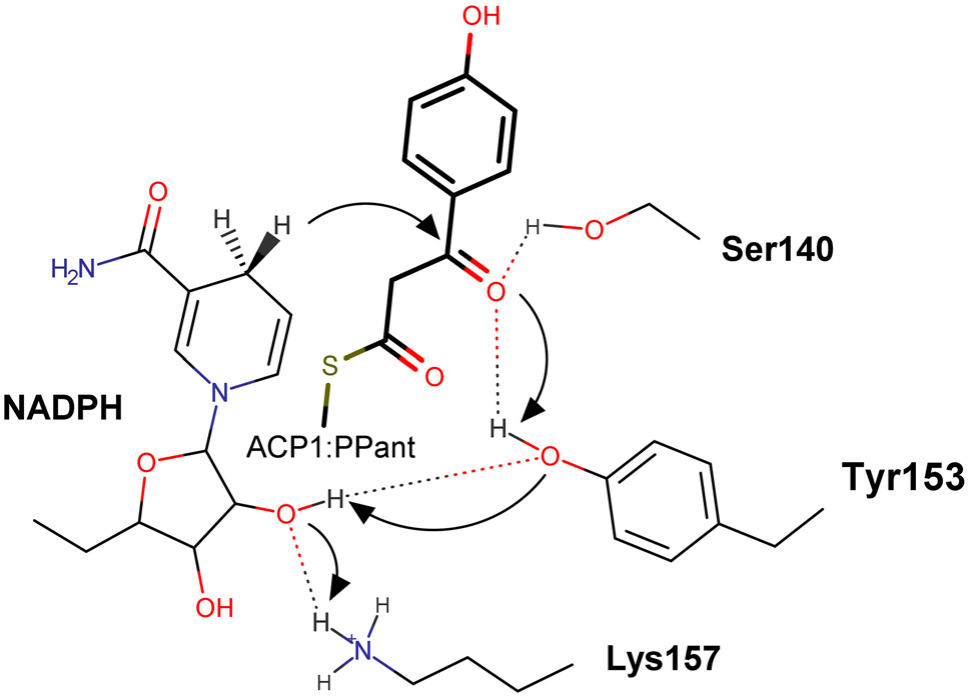
Proposed reaction scheme of ApeQ. Residues involved in the binding and catalysis of β-keto group reduction are presented. The visible moiety of the substrate is rendered as thicker bonds. Hydride transfer is depicted by arrows.

Based on our binding model of the substrate, we can propose a reaction scheme for ApeQ (Fig. 8). The β-carbon of the substrate is in the hydride transfer vicinity, and subsequent hydride relay from Tyr153 to Lys157 results in the *D*-hydroxy group in the substrate. As a conserved feature among SDR family members, the hydroxy group in the ribose ring of NADPH serves as a conduit to the electron transfer chain (Fig. 8).

The genomic structure of APE synthesis BGCs from Gram-negative bacteria comprises an inner membrane transporter, periplasmic lipid-binding protein, lipoprotein, glycosyltransferase, and two acyltransferases with no known function (Fig. 1a). The APE product may be further modified by these enzymes^14^ and then can be transported to the outer membrane. Identification of the role of these APE products in the evolution of GC2 CRAB strains may be important for tackling endemic GC2 strain infection. Recently, a red pigment from members of group B *Streptococcus* was identified as a hemolysin involved in host cell membrane penetration^42^. This pigment features a polyene group with 13 double bonds, in which each end is capped by ornithine and l-rhamnose. Penetration of the eukaryotic cell membrane is reminiscent of the effective actions of polyene macrolides against fungi^21^. Therefore, it will be of great interest to determine whether the final product of the *A. baumannii* APE BGC also plays a role in invasive host cell infection via penetrative action. Further studies regarding this final product will be required to elucidate the detailed function of APEs in Gram-negative pathogenic bacteria. The present mutagenesis experiment and ITC data confirmed that the unique surface electrostatic potential profiles near the positively charged arginine patch in ApeQ is the key feature for cognate ACP1 binding in APE biosynthesis. This specific information will facilitate the design of inhibitors for protein–protein interactions. Finally, we are currently performing structural analyses on cognate ACPs, ACP1 and ACP2, using a solution nuclear magnetic resonance technique. These upcoming data, combined with the present structural results on ApeQ, will help in elucidating the detailed mechanism underlying APE biosynthesis and provide new insights for its effective control.

## Methods

### Cloning of APE genes and AbFabG

All genes analyzed in this study were PCR-amplified from the genomic DNA of a clinically isolated CRAB strain designated C1. Genomic DNA was extracted from an overnight culture of *A. baumannii* [grown in Luria–Bertani (LB) medium] using a bacteria mini kit (Cosmogenetech, Seoul, Republic of Korea) following standard procedures. Primer sets were designed based on the *A. baumannii* ACICU genomic DNA sequence. All primer sequences used in this study are listed in Supplementary Table S1. The PCR products of AbApeQ and AbFabG were cut using the *Nde*I and *Xho*I restriction enzymes and ligated into the pET28a vector (Novagen, Madison, WI, USA) to produce recombinant proteins with an N-terminal His-tag and thrombin site. The recombinant ACP1 and ACP2 were then cloned into the pET21a vector with the same set of restriction enzymes to generate clones without the His-tag. *A. baumannii* FabD enzyme was cloned as previously described^18^. *A. baumannii* holo-ACP synthase (AbAcpS) was prepared as previously described^17^.

For co-expression of heterodimeric enzymes, the ApeO/ApeC (CLF-KS) and ApeH/ApeP dehydratase genes were ligated into two multiple cloning sites (MCS1 and MCS2) of the pETDuet-1 vector. ApeO and ApeH were amplified by primers containing the *Bam*HI and *Xho*I restriction sites to be placed at the *Bam*HI/*Sal*I sites of the pETDuet-1 MCS1 position. The recombinant proteins contained an N-terminal His-tag. ApeO and ApeP were amplified by primers containing *Nde*I and *Sal*I to be placed at the *Nde*I/*Xho*I sites of MCS2 in the pETDuet-1 vector (Supplementary Table S1).

### Expression and purification

The expression vectors were transformed into *E. coli* BL21 (DE3) and grown in LB media at 37 °C until the optical density (OD) at 600 nm reached 0.8, at which point 0.2 mM isopropyl-β-d-thiogalactopyranoside (IPTG) was added and the cells were further incubated at 25 °C overnight. The cells were harvested by centrifugation and stored at − 80 °C until use. The cells were resuspended in binding buffer (20 mM Tris pH 8 and 100 mM NaCl) and homogenized by sonication. Cell debris was removed by centrifugation at 13,000 rpm and the cleared lysate was loaded onto a Chelating Sepharose FF 5-mL column (GE Healthcare, Piscataway, NJ, USA). Bound proteins were eluted by gradual elution with 20 mM Tris (pH 8), 100 mM NaCl, and 500 mM imidazole. Fractions containing the proteins of interest were pooled and further purified by a Q-Sepharose HiTrap HP 5-mL anion exchange chromatography column with an NaCl gradient of 0–1 M in 20 mM Tris buffer (pH 8) (GE Healthcare). Apo ACP1 and ACP2 were purified using Q-Sepharose anion-exchange chromatography and Superdex 200 size-exclusion chromatography. The purified proteins were buffer-exchanged and concentrated using 20 mM Tris (pH 8) and 100 mM NaCl storage buffer by an Amicon filter (GE Healthcare).

AbApeQ and AbFabG were incubated with thrombin (Haematologic, Essex Junction, VT, USA) to remove the His-tag prior to anion-exchange chromatography. The eluted fractions were pooled and buffer-exchanged using the storage buffer containing 20 mM Tris (pH 8) and 100 mM NaCl. The protein concentration was quantified by measuring the absorption at 280 nm using a NanoDrop spectrophotometer (Thermo Fisher Scientific, Waltham, MA, USA). The molar concentration of the protein was adjusted using the absorption coefficient estimated using the ExPasy server^43^.

For the generation of holo-ACPs, ACP1 and ACP2 were co-expressed with AbAcpS as previously described^17^. In brief, either the ACP1 or ACP2 gene was cloned into the *Nde*I/*Xho*I sites of MCS2 in the pETDuet-1 vector containing the AbAcpS sequence in MCS1 to generate two expression vectors: holo-ACP1 and holo-ACP2. Each expression vector was transformed into *E. coli* BL21 (DE3) cells and grown in LB medium at 37 °C until the OD at 600 nm reached 0.8 before induction with 0.2 mM IPTG for 2 h. The cells were harvested and resuspended in 20 mM Tris (pH 8), 100 mM NaCl, and 2 mM dithiothreitol (DTT). ACP1 and ACP2 were purified by Q-Sepharose (5 mL) anion-exchange chromatography and Superdex 75 size-exclusion chromatography. Apo-ACP proteins were removed by Resource Q 5-mL anion exchange chromatography (GE Healthcare); these were verified by native polyacrylamide gel electrophoresis.

The F185L, R148A, and R174A mutants of AbApeQ were prepared for biochemical characterization by PCR using DyeMix Forte Pfu polymerase (Enzynomics, Daejon, Korea). The expression vectors were transformed into *E. coli* BL21 (DE3) cells and purified as described above for AbApeQ or AbFabG. The reaction was followed by UV/Vis absorption spectroscopy, as described for wild-type proteins.

### Crystallization and structure determination

AbApeQ was crystallized using the PEG Ion screen kit (Hampton Research, USA). Crystals appeared several days after crystallization with the crystallization buffer containing 50 mM HEPES pH 7, 12% (w/v) PEG 3350, and 1% (w/v) tryptone. For crystallization of the complex of the protein with NADPH, a 1:3 molar ratio of NADPH sodium salt (Sigma Aldrich, St. Louis, MO, USA) was added to the protein solution, followed by incubation on ice for 1 h before crystallization. Crystals were observed following addition of the crystallization buffer, which was composed of 0.2 M potassium sodium tartrate and 20% (w/v) PEG 3350. Apo AbFabG crystals were obtained using crystallization condition of 0.1 M sodium formate pH 7.0 and 12% (w/v) PEG 3350.

Prior to data collection, the crystals were immersed in the crystallization buffer supplemented with 25% (v/v) ethylene glycol to prevent ice formation and then flash-cooled with liquid nitrogen. X-ray diffraction data sets were collected using the beamline 7A system at the Pohang Accelerator Laboratory at a wavelength of 0.975 Å. Diffraction images were integrated and scaled using the XDS program^44^. The structure of AbApeQ was determined by molecular replacement using the coordinates of a putative FabG structure from *E. coli* CFT073 (PDB ID: 4IIU) with the program Phaser^45^. Modeling and refinement of the AbApeQ structures were performed using the programs Coot and Phenix, respectively^46,47^. The data collection and refinement statistics are listed in Supplementary Table S2. The three-dimensional structural coordinates of the structures have been deposited in the Protein Data Bank under accession numbers: 7CAW (apo AbApeQ), 7CAX (AbApeQ-NADPH complex) and 7CAZ (apo AbFabG).

### In vitro APE biosynthesis assay

To assess the contribution of mutagenesis on APE biosynthesis by *A. baumannii*, the UV/Vis absorption spectrum of either NADPH as the co-substrate (absorbance at 340 nm) or APE-ACP1 as the product (absorbance at ~ 450 nm) was measured in the range of 300–600 nm. Prior to the measurement, benzoyl-ACP1 was generated by the coupling of 5 mM ATP and 1.93 mM holo-ACP1 in the presence of 4-hydroxybenzoic acid. Malonyl-ACP was generated in situ by the addition of malonyl-CoA and AbFabD in the 200-μL reaction mixture. The enzymatic assay was conducted using 2 mM DTT, 100 μM of benzoyl-ACP1, 1.17 mM of malonyl-CoA, 13 μM of holo-ACP2, 300 μM of NADPH, and 5 μM each of FabD, ApeC/ApeO, ApeH/ApeP, and the ketoreductases AbApeQ and AbFabG. Baseline activities were measured after mixing benzoyl-ACP1, holo-ACP2, and malonyl-CoA. The reaction was initiated by adding NADPH and enzymes, including ketoreductases, and the spectra spanning 300–600 nm were recorded every 60 s for a total of 15 min using the Optizen Pop UV/VIS spectrophotometer (Optizen, Daejeon, Republic of Korea) at room temperature (25 °C). From the spectra, a decrease in the levels of NADPH (340 nm) and an increase in the levels of APE products (460 nm) were compared to obtain the relative conversion rate.

### ITC

ITC was conducted using MicroCal 200 (Malvern Panalytical Inc., USA). For ITC, we prepared AbFabG, AbApeQ, and its variants in the final concentration of 0.1 mM. The ligand holo ACP1 was concentrated to 2 mM. Both samples were in buffer containing 20 mM Tris pH 8, 100 mM NaCl, and 2 mM NADPH. All the procedures were conducted at 25 °C. All results were analyzed using MicroCal PEAQ-ITC analysis software (ver. 1.22).

### Substrate binding model of ApeQ

To generate the coordinates for the substrate fragment, GRADE Web Server (Global Phasing Ltd, Cambridge, UK) was used with default settings^48^. The molecular model was used without modification and was fit into the active site using the restraints described below. We used Maestro software version 12.4 (Schrodinger Inc., USA) to dock the fragment with constraints on two residues, namely Asp93 and Gln150, to form hydrogen bonds with the ligand. The ligand and protein coordinates were generated using forcefield OPLS3e. The AbApeQ structure was prepared by removing water and used without energy minimization. The receptor grid was placed in the vicinity of the nicotinamide hydride transfer. Docking simulations were run using Glide subroutine in standard precision mode. Post minimization was performed on the ligand to optimize bond lengths and torsion angles.

## Supplementary Information

Supplementary Information.

## Data Availability

The three-dimensional structural coordinates of the structures have been deposited in the Protein Data Bank under accession numbers: 7CAW (apo AbApeQ), 7CAX (AbApeQ-NADPH complex) and 7CAZ (apo AbFabG).

## Abbreviations

FAS: Fatty acid synthase
ACP: Acyl carrier protein
KAS: Ketoacyl-acyl carrier protein synthase
PKS: Polyketide synthase
APE: Aryl polyene
BGC: Biosynthesis gene cluster
CLF-KS: Chain length factor-β-ketosynthase
AasS: Acyl-ACP synthetase
MCAT: Malonyl-CoA transacylases
MDR: Multi-drug resistant
CRAB: Carbapenem-resistant *A. baumannii*
OXA: Oxacillinase
SDR: Short-chain dehydrogenase/reductase
RMSD: Root mean square deviation
Ppant: Phosphopantetheine
