# Supplementary material for: Structural comparison of Acinetobacter baumannii β-ketoacyl-acyl carrier protein reductases in fatty acid and aryl polyene biosynthesis
Source: Sci Rep. 2021 Apr 12;11:7945. doi: 10.1038/s41598-021-86997-3 (PMC8041823; doi:10.1038/s41598-021-86997-3)
Supplement: Supplementary file 1 — Supplementary Information. [file 41598_2021_86997_MOESM1_ESM.pdf]

Supporting Information

Structural comparison of *Acinetobacter baumannii* β-ketoacyl-acyl carrier protein reductases in fatty acid and aryl polyene biosynthesis

Woo Cheol Lee, Sungjae Choi, Ahjin Jang, Kkabi Son, Yangmee Kim*

Department of Bioscience and Biotechnology, Konkuk University, Seoul 05029, Republic of Korea

***Corresponding Author**

Yangmee Kim, email: ymkim@konkuk.ac.kr

Supplementary Figure S1. Phylogenetic tree of strains GC1 and GC2.

Supplementary Figure S2. Genomic context of a FAS gene cluster including FabG among nosocomial pathogenic *A. baumannii* strains.

Supplementary Figure S3. Comparison of the genomic context for a representative strain of GC1 (*A. baumannii* AYE) and GC2 (*A. baumannii* ACICU).

Supplementary Figure S4. Size exclusion chromatography elution profile of AbApeQ.

Supplementary Figure S5. SDS-PAGE of proteins used in the study.

Supplementary Figure S6. Time course plot of APE synthesis as observed at 460 nm (A_{460}).

Supplementary Figure S7. The superposed ribbon models of AbApeQ and AbFabG

Supplementary Table S1. PCR primers used in this study.

Supplementary Table S2. X-ray crystallography data collection and refinement statistics.

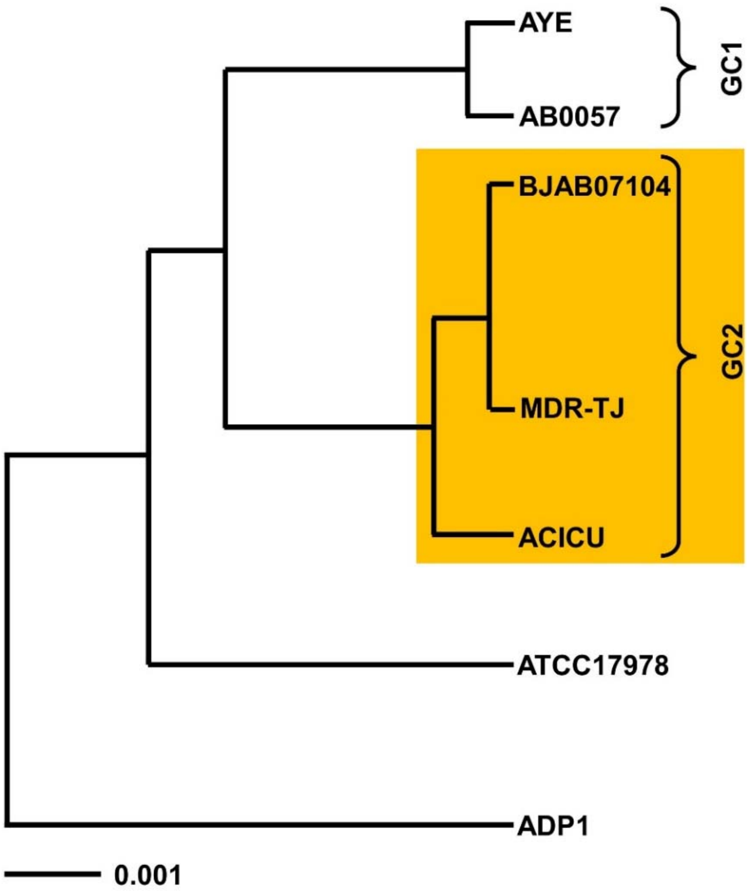

Supplementary Figure S1. Phylogenetic tree of strains GC1 and GC2. *A. baumannii* strains containing the APE BGC are highlighted in the orange box.

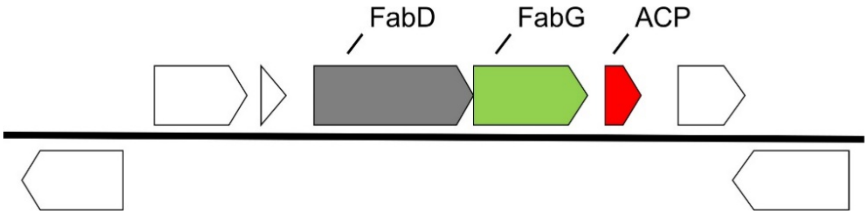

Supplementary Figure S2. Genomic context of a FAS gene cluster including FabG among nosocomial pathogenic *A. baumannii* strains.

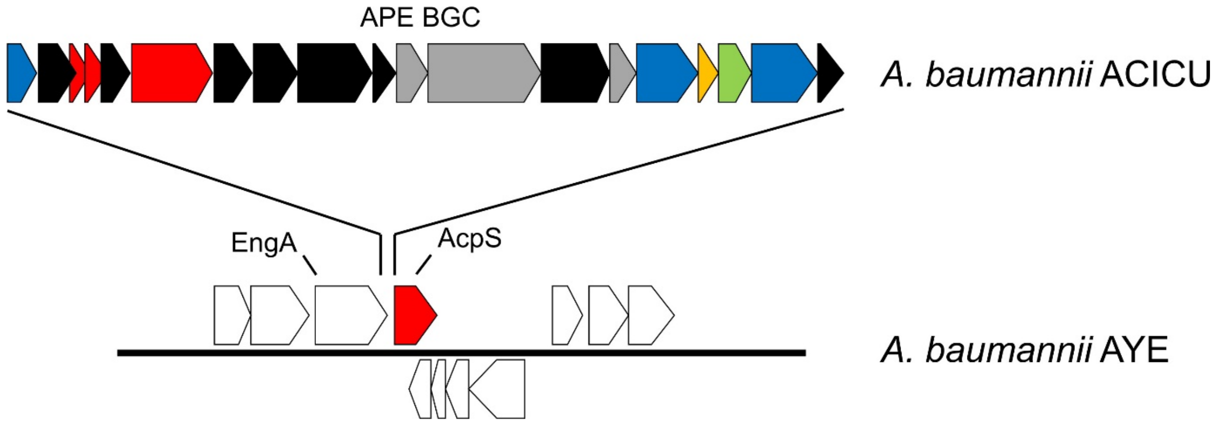

Supplementary Figure S3. Comparison of the genomic context for a representative strain of GC1 (*A. baumannii* AYE) and GC2 (*A. baumannii* ACICU). The color scheme is the same as that described in **Figure 1**.

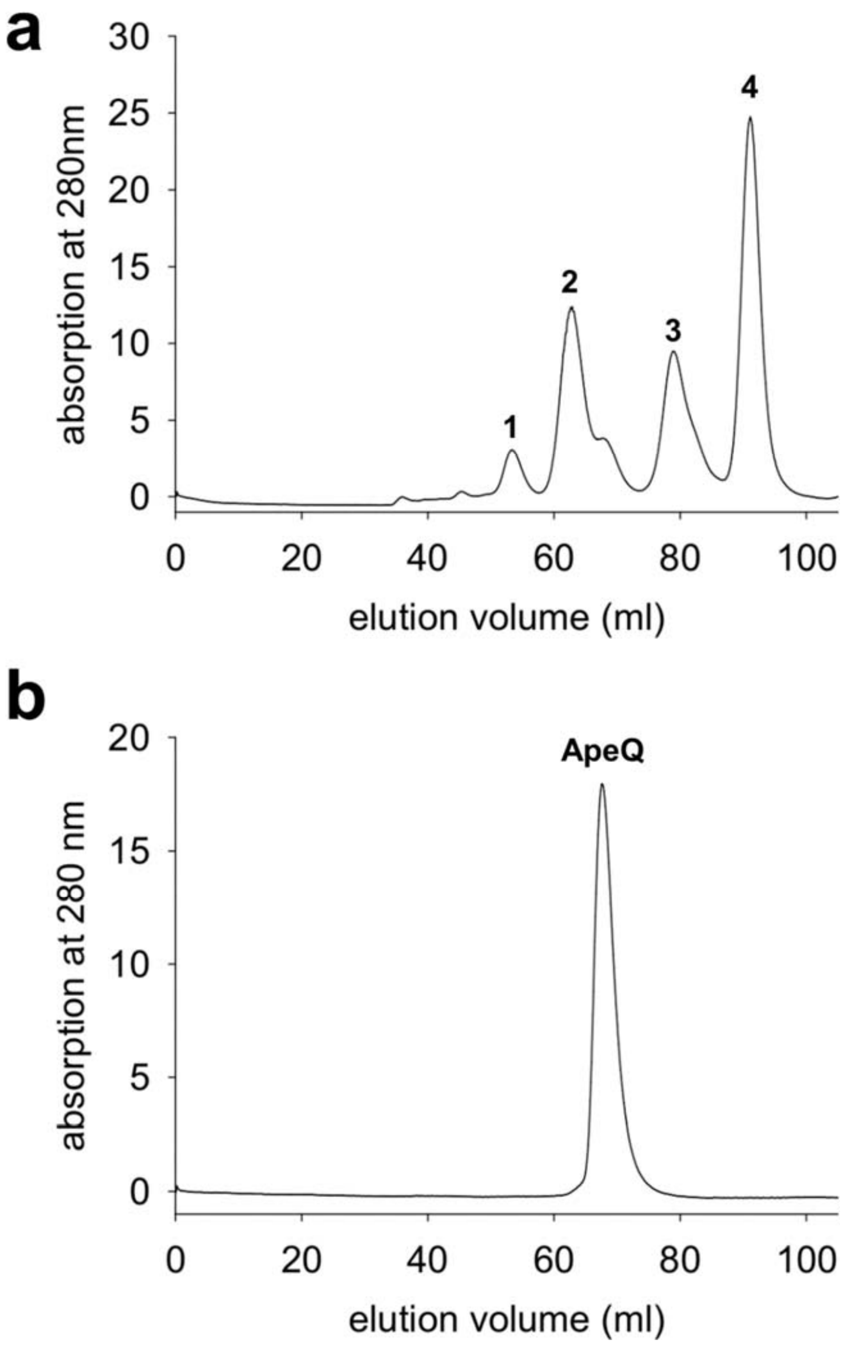

Supplementary Figure S4. Size exclusion chromatography of AbApeQ using a Superdex 200 16/60, prep. grade column (GE Healthcare) with 20 mM Tris HCl pH 8, 100 mM NaCl buffer. **a**. Run with gel filtration protein standards (Bio-Rad, USA): **1**, thyroglobulin (bovine, molecular weight (MW) = 670000, eluted at 53.3 ml); **2**. γ-globulin (bovine, MW = 158000, 63.1 ml); **3**. ovalbumin (chicken, MW = 44000, 78.8 ml); **4**. myoglobin (horse, MW = 17000, 91.1 ml). **b**. Run with purified ApeQ. The eluted volume of 67.2 mL corresponds to a molecular weight of 142 kDa (R^2 = 0.9791), larger than the calculated tetrameric molecular weight (105.6 kDa) of AbApeQ.

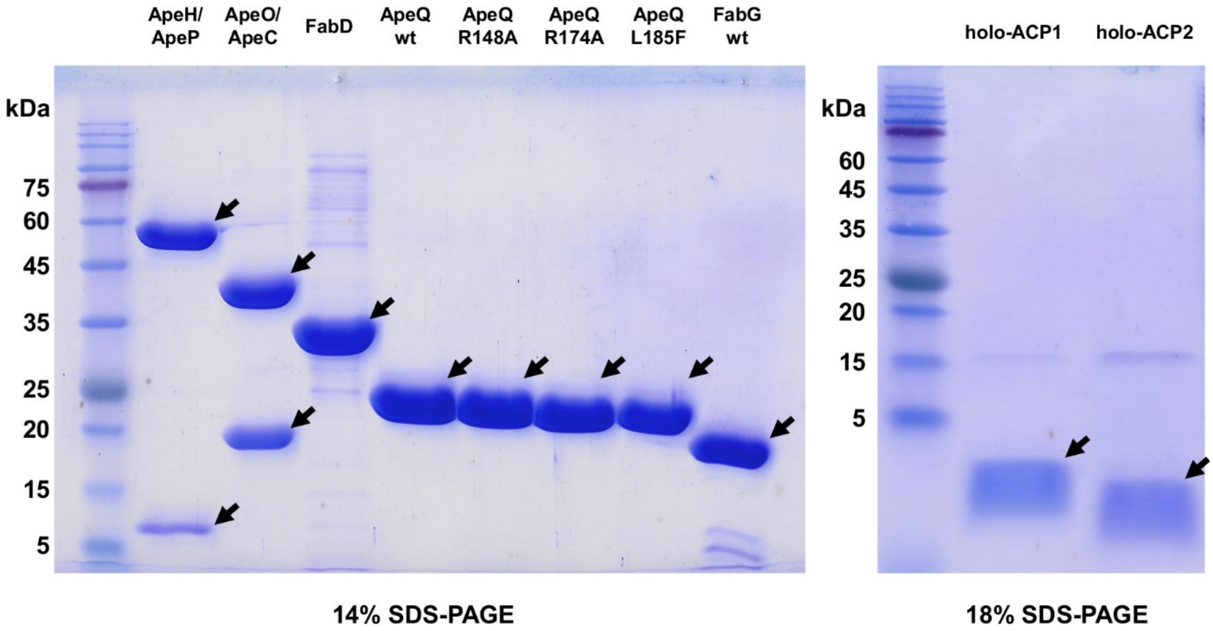

Supplementary Figure S5. SDS-PAGE of proteins used in this study. Approximately 4 µg of each protein (complex) was loaded onto 14% or 18% SDS-PAGE gel.

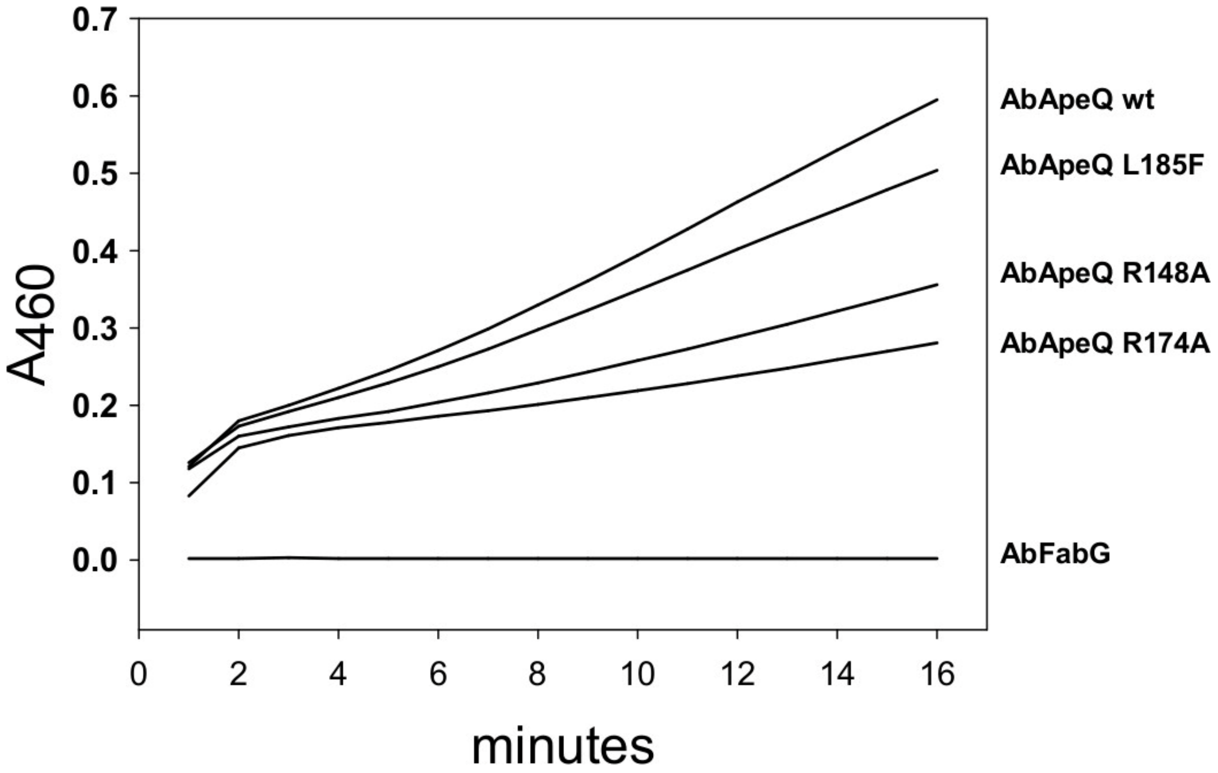

Supplementary Figure S6. Time course plot of APE synthesis in vitro as observed at 460 nm.

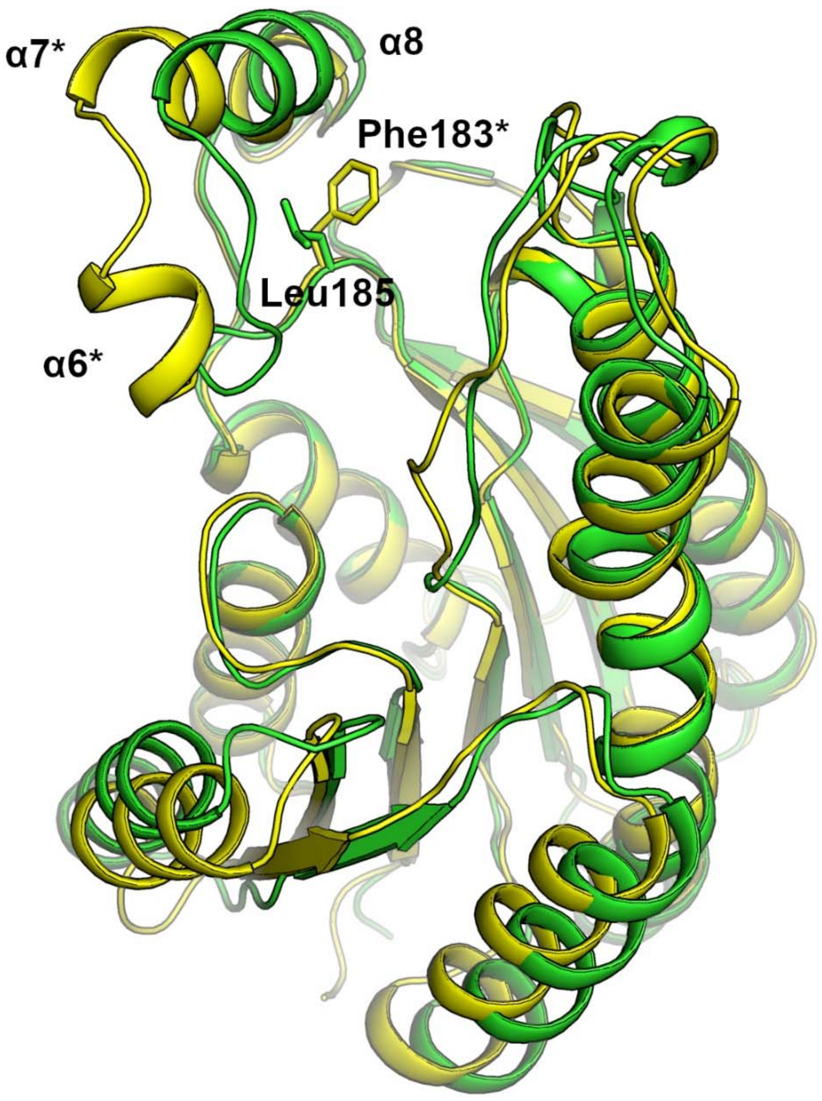

Supplementary Figure S7. The superposed ribbon models of AbApeQ (green) and AbFabG (yellow). A residue or secondary structure elements of AbFabG are designated with asterisks.

Protein	Primer	Sequence (5’-3’)
AbFabG	sense	CGCATATGACACAAGAACGCAAAGTTGC
	antisense	CGCTCGAGTTAGGCCATGTATAAACCACC
AbApeQ	sense	CGCATATGACAAGACGAATTTTAGTAACG
	antisense	CGCTCGAGTCAGATGAGTCCTCCATTC
AbACP1	sense	GCCCATGGATGAAGAGTTATCTTGAAATGAGC
	antisense	GCGGATCCTTATGACTTCTGTTGTGCTTC
AbACP2	sense	GCCATATGTTATCTCAAGAGCAAGTATTAAC
	antisense	GCCTCGAGTCATTCAGCAGTCATGTTCTG
AbFabD	sense	CGCATATGATGTCTGCTAAACGTCTTGAACAAG
	antisense	CGGGATCCTCATGCAATTTTTCCTTCTGCCAC
AbApeH	sense	CGGGATCCGTATTGCCATTTTCAGAAATTC
	antisense	CGCTCGAGTTAATCCTCTACTTCTGTTTG
AbApeC	sense	CGCATATGGTAACACTACATCTTGCTCATTTAAC
	antisense	CGCTCGAGTTAGCATTTGTTCCACCCAAATGCAG
AbApeK	sense	CGCATATGCATGCAGATGTAATCATTG
	antisense	CGCTCGAGTCACGCATTCCTCTTCGGTTG
AbApeO	sense	CGGGATCCGAAACATTTACCTTCAGAAAA
	antisense	CGCTCGAGTTAGGGCTTCACTCCAAAAACAAGAC
AbAcpS	sense	CGGGATCCGGTTCGGATTAATAAAATGAATAGTAAAC
	antisense	CGGTCGACTTAACGGTCAAGTGAATGAATCTG
AbApeQ L185F	sense	GTGTAGCTCCTGGATTTATCGAAACCGAAATG
	antisense	CATTTCGGTTTCGATAAATCCAGGAGCTACAC
AbApeQ R148A	sense	GGAATTATGGGTAACGCTGGTCAGGTTAAC
	antisense	GTTAACCTGACCAGCGTTACCCATAATTCC
AbApeQ R174A	sense	CTCGAATTGGCGAAGGCAAAAATTACTGTGAAC
	antisense	GTTCACAGTAATTTTTGCCTTCGCCAATTCGAG

Supplementary Table S1. PCR primers used in this study. Restriction sites are underlined.

	apo AbApeQ	AbApeQ-NADPH	apo AbFabG
**Data collection**			
PDB ID	7CAW	7CAX	7CAZ
Resolution (Å)	1.88	1.85	1.79
Space group	$P3_121$	$P2_1$	$P2_12_12_1$
Cell constants	a = b = 89.60, c = 239.77 Å	a = 61.93, b = 90.02, c = 97.78 (Å), β = 9 2.7°	a = 78.61, b = 100.0 4, c = 120.17 Å
R_{merge}	0.05	0.08	0.07
I/σI	16.6 (5.5)	9.4 (2.0)	13.6 (2.1)
Redundancy	2.83	1.9	4.62
Unique reflections	165899	332397	88090
Completeness (%)	94	96.4	98.2
Wilson B-factor	16.4	22.8	19.8
**Refinement**			
R-factor	0.160	0.172	0.207
free-*R*	0.191	0.199	0.229
RMSD			
Bond lengths (Å)	0.007	0.007	0.010
Bond angles (°)	0.763	0.818	1.195
Overall *B*-factor	17.0	25.0	29.0
Ramachandran plot			
Favored (%)	92.4	92.1	92.9
Allowed (%)	7.6	7.9	6.6
Generously allowed (%)	0	0	0.5
Disallowed (%)	0	0	0

Supplementary Table S2. X-ray crystallography data collection and refinement statistics.

Note: Values in parentheses are of the outermost shells.
